# Supplementary material for: Molecular Variability of the Fusarium solani Species Complex Associated with Fusarium Wilt of Melon in Iran
Source: J Fungi (Basel). 2023 Apr 18;9(4):486. doi: 10.3390/jof9040486 (PMC10142084; doi:10.3390/jof9040486)
Supplement: Supplementary file 1 [file jof-09-00486-s001.zip › Figure S4.pdf]

Figure S4

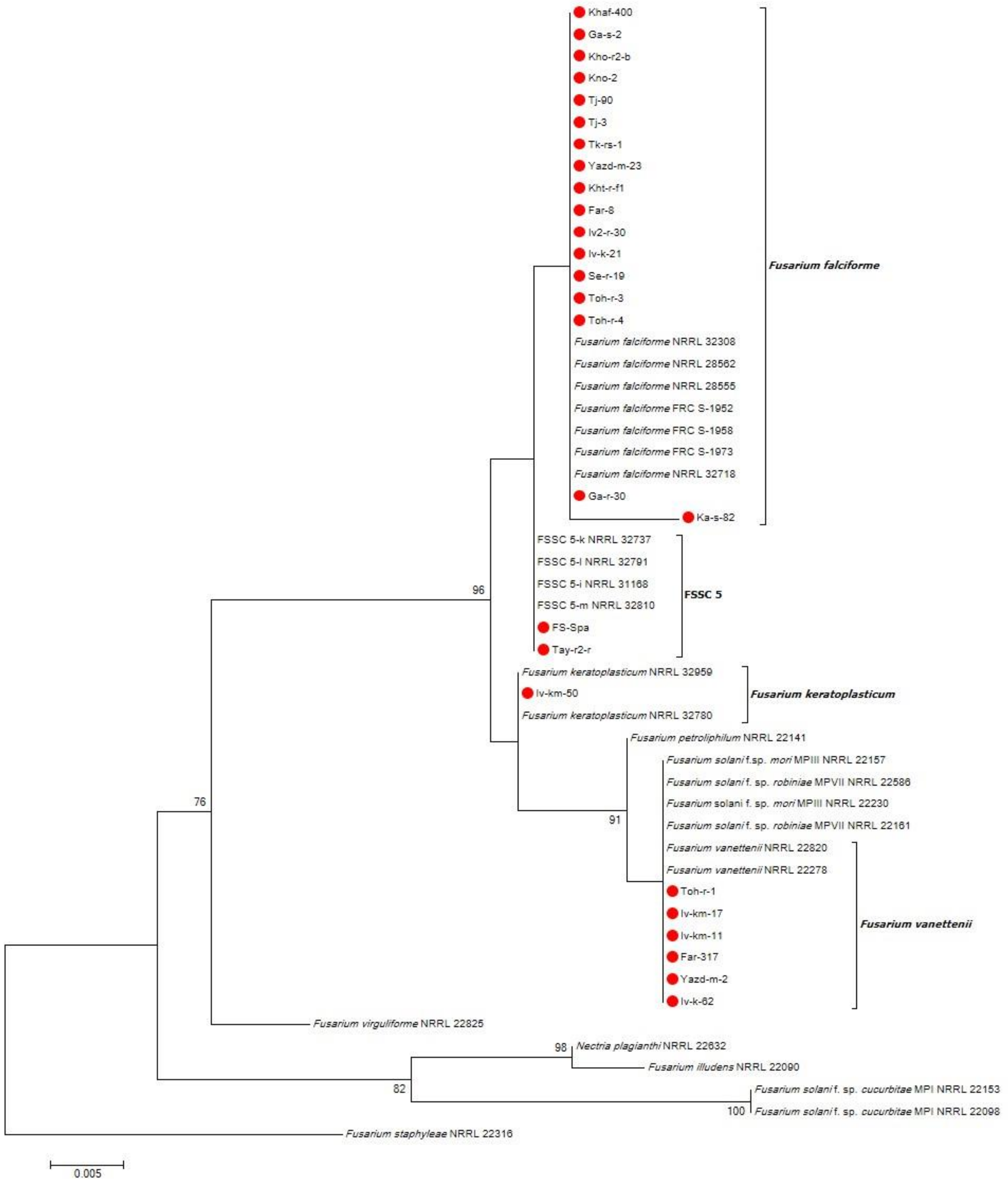

Khaf-400
Ga-s-2
Kho-r2-b
Kno-2
Tj-90
Tj-3
Tk-rs-1
Yazd-m-23
Kht-r-f1
Far-8
Iv2-r-30
Iv-k-21
Se-r-19
Toh-r-3
Toh-r-4
Fusarium falciforme NRRL 32308
Fusarium falciforme NRRL 28562
Fusarium falciforme NRRL 28555
Fusarium falciforme FRC S-1952
Fusarium falciforme FRC S-1958
Fusarium falciforme FRC S-1973
Fusarium falciforme NRRL 32718
Ga-r-30
Ka-s-82
Fusarium falciforme
FSSC 5-k NRRL 32737
FSSC 5-l NRRL 32791
FSSC 5-i NRRL 31168
FSSC 5-m NRRL 32810
FS-Spa
Tay-r2-r
FSSC 5
96
Fusarium keratoplasticum NRRL 32959
Iv-km-50
Fusarium keratoplasticum NRRL 32780
Fusarium keratoplasticum
Fusarium petroliphilum NRRL 22141
Fusarium solani f.sp. mori MPIII NRRL 22157
Fusarium solani f. sp. robiniae MPVII NRRL 22586
Fusarium solani f. sp. mori MPIII NRRL 22230
Fusarium solani f. sp. robiniae MPVII NRRL 22161
76
91
Fusarium vanettenii NRRL 22820
Fusarium vanettenii NRRL 22278
Toh-r-1
Iv-km-17
Iv-km-11
Far-317
Yazd-m-2
Iv-k-62
Fusarium vanettenii
Fusarium virguliforme NRRL 22825
98
Nectria plagianthi NRRL 22632
Fusarium illudens NRRL 22090
82
Fusarium solani f. sp. cucurbitae MPI NRRL 22153
100
Fusarium solani f. sp. cucurbitae MPI NRRL 22098
Fusarium staphyleae NRRL 22316
0.005
