# Supplementary material for: Molecular Variability of the Fusarium solani Species Complex Associated with Fusarium Wilt of Melon in Iran
Source: J Fungi (Basel). 2023 Apr 18;9(4):486. doi: 10.3390/jof9040486 (PMC10142084; doi:10.3390/jof9040486)
Supplement: Supplementary file 1 [file jof-09-00486-s001.zip › Table S1.pdf]

Table S1

Species	Isolate	Country	Year	Source	Accession numbers		
					ITS	LSU	*tef1*
F. falciforme	Se-r-19	Iran	2010	long melon	**OQ396678**	**OQ308937**	**OP957290**
	Khaf-400	Iran	2009	long melon	**OQ396679**	**OQ308938**	**OP957291**
	Toh-r-3	Iran	2009	long melon	**OQ396680**	**OQ308939**	**OQ428629**
	Toh-r-4	Iran	2009	long melon	**OQ396681**	**OQ308940**	**OP957296**
	Iv-k-21	Iran	2011	long melon	**OQ396682**	**OQ308941**	**OP957292**
	Iv2-r-30	Iran	2009	long melon	**OQ396683**	**OQ308942**	**OQ428630**
	Yazd-m-23	Iran	2010	cantaloupe	**OQ396684**	**OQ308943**	**OP957295**
	Tj-90	Iran	2009	long melon	**OQ396685**	**OQ308944**	**OQ428642**
	Tk-rs-1	Iran	2011	cantaloupe	**OQ396686**	**OQ308945**	**OQ428643**
	Ga-r-30	Iran	2009	cantaloupe	**OQ396687**	**OQ308946**	**OQ428631**
	Ga-s-2	Iran	2011	cantaloupe	**OQ396688**	**OQ308947**	**OQ428632**
	Ka-s-82	Iran	2010	long melon	**OQ396689**	**OQ308948**	**OQ428633**
	Tj-3	Iran	2010	long melon	**OQ396690**	**OQ308949**	**OQ428645**
	Far-8	Iran	2011	long melon	**OQ396691**	**OQ308950**	**OP957297**
	Kht-r-f1	Iran	2009	long melon	**OQ396692**	**OQ308951**	**OQ428644**
	Kno-2	Iran	2009	cantaloupe	**OQ396693**	**OQ308952**	**OP957293**
	Kho-r2-b	Iran	2011	cantaloupe	**OQ396694**	**OQ308953**	**OP957294**
	NRRL 32718	USA	2006	human eye	DQ094500	DQ236542	DQ247038
	FRC S-1973	Aust	2011	soil	JF433073	JF433073	DQ247494
	FRC S-1958	Aust	2011	soil	JF433064	JF433064	DQ247481
	FRC S-1952	Aust	2011	soil	JF433060	JF433060	DQ247487
	NRRL 28555	USA	2006	human wrist	DQ094369	DQ236411	DQ246896
	NRRL 28562	USA	2006	human bone	DQ094376	DQ236418	DQ246903
	NRRL 32308	SA	2006	Human foot	DQ094406	DQ236448	DQ246936
F. illudens	NRRL 22090	USA	ND	ND	AF178393	AF178362	AF178326
F. keratoplasticum	Iv-km-50	Iran	2009	long melon	**OQ396703**	**OQ308962**	**OP957298**
	NRRL 32780	USA	2006	Sea turtle	DQ094551	DQ236593	DQ247090
	NRRL 32959	USA	2006	Human skin	DQ094632	DQ236674	DQ247178
F. petroliphilum	NRRL 22141	USA	2007	ND	DQ094307	DQ236349	AF178329
F. plagianthi	NRRL 22632	USA	1999	ND	AF178417	AF178386	AF178354
	NRRL 22098	USA	2005	ND	DQ094301	DQ236343	AF178327
F. solani f. sp. *cucurbitae* MPI	NRRL 22153	USA	1999	ND	AF178410	AF178379	AF178346
F. solani f. sp. *mori* MPIII	NRRL 22157	USA	1999	ND	AF178421	AF178390	AF178359

	NRRL 22230	USA	1999	ND	AF178420	AF178389	AF178358
F. solani f. sp. *robiniae*	NRRL 22161	USA	2005	Rubinea	DQ094311	DQ236353	AF178330
MPVII	NRRL 22586	USA	2005	Rubinea	DQ094312	DQ236354	AF178353
FSSC 5	Tay-r2-r	Iran	2009	long melon	**OQ396695**	**OQ308954**	**OQ428640**
	FS-Spa	Spain	ND	ND	**OQ396696**	**OQ308955**	**OQ428641**
	NRRL 31168	USA	2006	human toe leukemia	DQ094395	DQ236437	DQ246922
	NRRL 32810	USA	2006	Human eye	DQ094577	DQ236619	DQ247118
	NRRL 32737	USA	2006	Human eye	DQ094518	DQ236560	DQ247057
	NRRL 32791	USA	2006	human	DQ094560	DQ236602	DQ247100
F. staphyleae	NRRL 22316	USA	1999	ND	AF178423	AF178392	AF178361
F. vanettenii	Iv-k-62	Iran	2009	long melon	**OQ396697**	**OQ308956**	**OQ428634**
	Far-317	Iran	2009	long melon	**OQ396698**	**OQ308957**	**OQ428635**
	Iv-km-11	Iran	2010	long melon	**OQ396699**	**OQ308958**	**OQ428636**
	Iv-km-17	Iran	2010	long melon	**OQ396700**	**OQ308959**	**OQ428637**
	Toh-r-1	Iran	2009	long melon	**OQ396701**	**OQ308960**	**OQ428638**
	Yazd-m-2	Iran	2010	long melon	**OQ396702**	**OQ308961**	**OQ428639**
	NRRL 22820	USA	ND	ND	DQ094310	DQ236352	AF178355
	NRRL 22278	USA	ND	ND	DQ094309	DQ236351	AF178337
F. virguliforme	NRRL 22825	USA	1999	ND	GU170655	AF178388	AF178357

ND: not determined; Aust: Australia; SA: Saudi Arabia

GenBank accessions of the strains from this study are indicated in bold.

Fusarium staphyleae was used as the outgroup.
