# Supplementary figures and images for: Molecular Variability of the Fusarium solani Species Complex Associated with Fusarium Wilt of Melon in Iran
Source: J Fungi (Basel). 2023 Apr 18;9(4):486. doi: 10.3390/jof9040486 (PMC10142084; doi:10.3390/jof9040486)

Figure S1

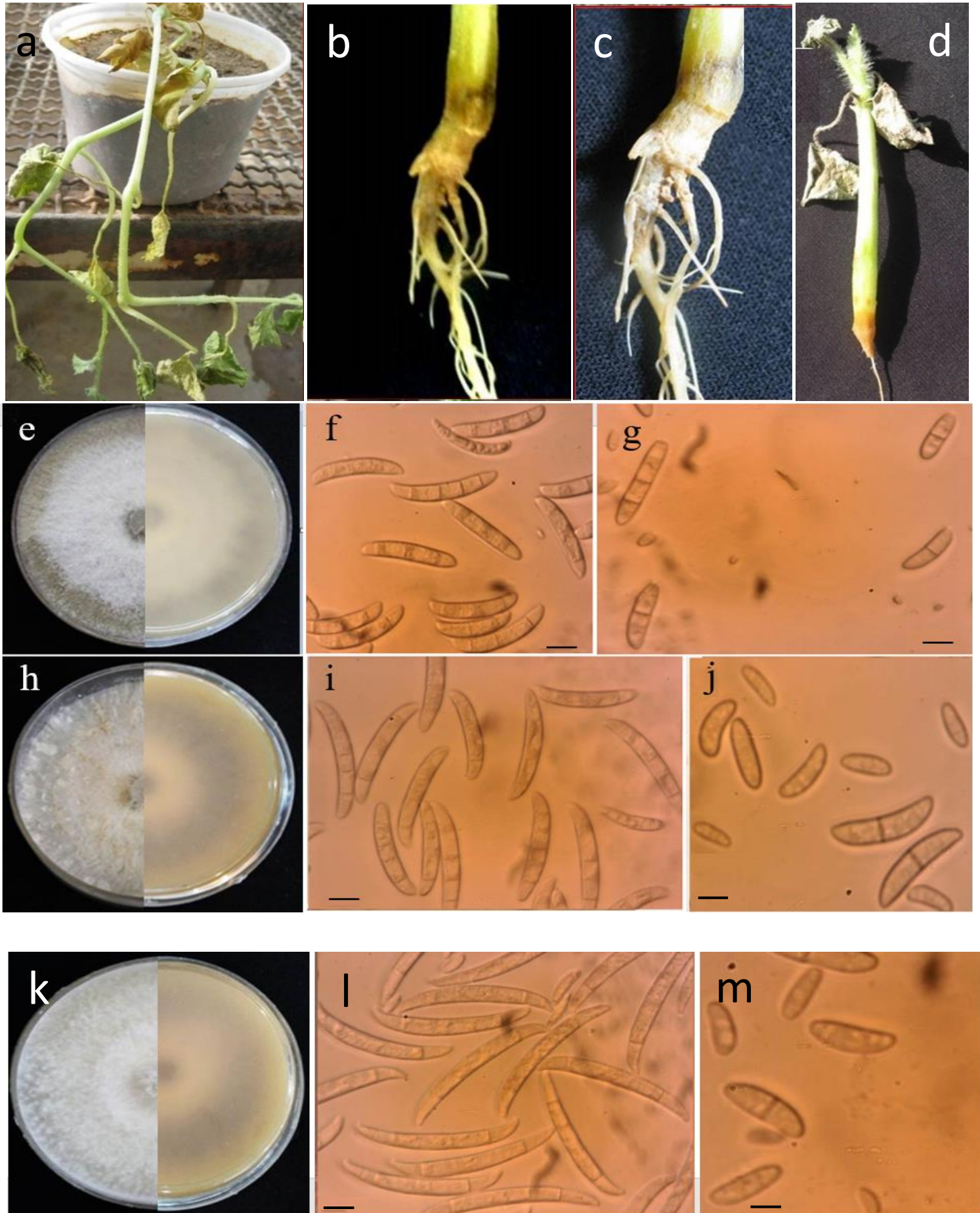

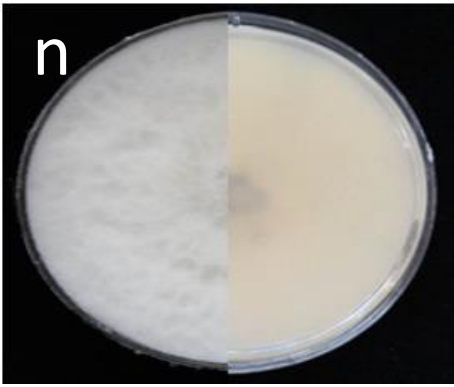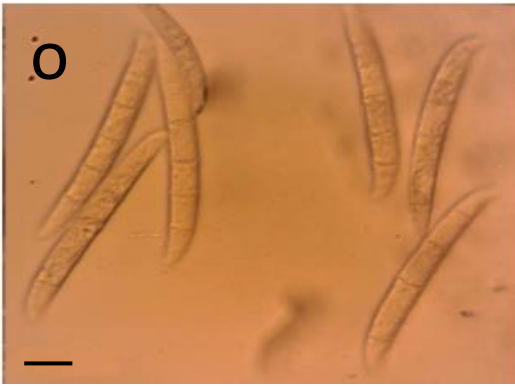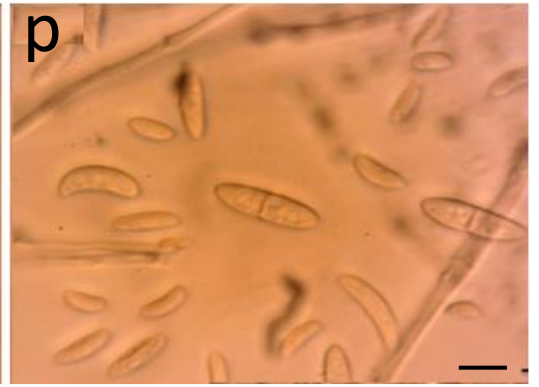

Supplement: Supplementary file 1 [file jof-09-00486-s001.zip › Figure S1.pdf]

Figure S2

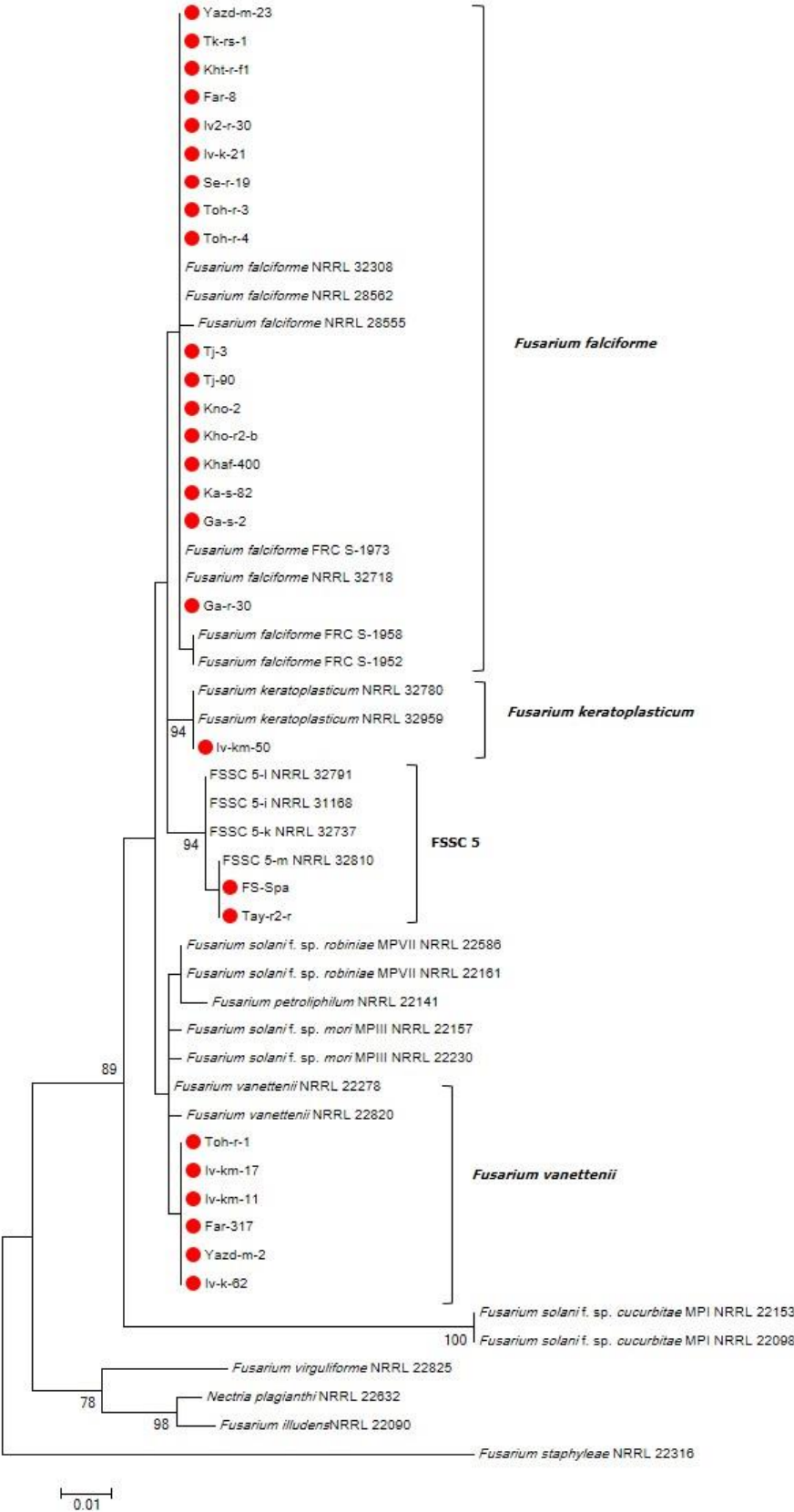

Supplement: Supplementary file 1 [file jof-09-00486-s001.zip › Figure S2.pdf]

Figure S3

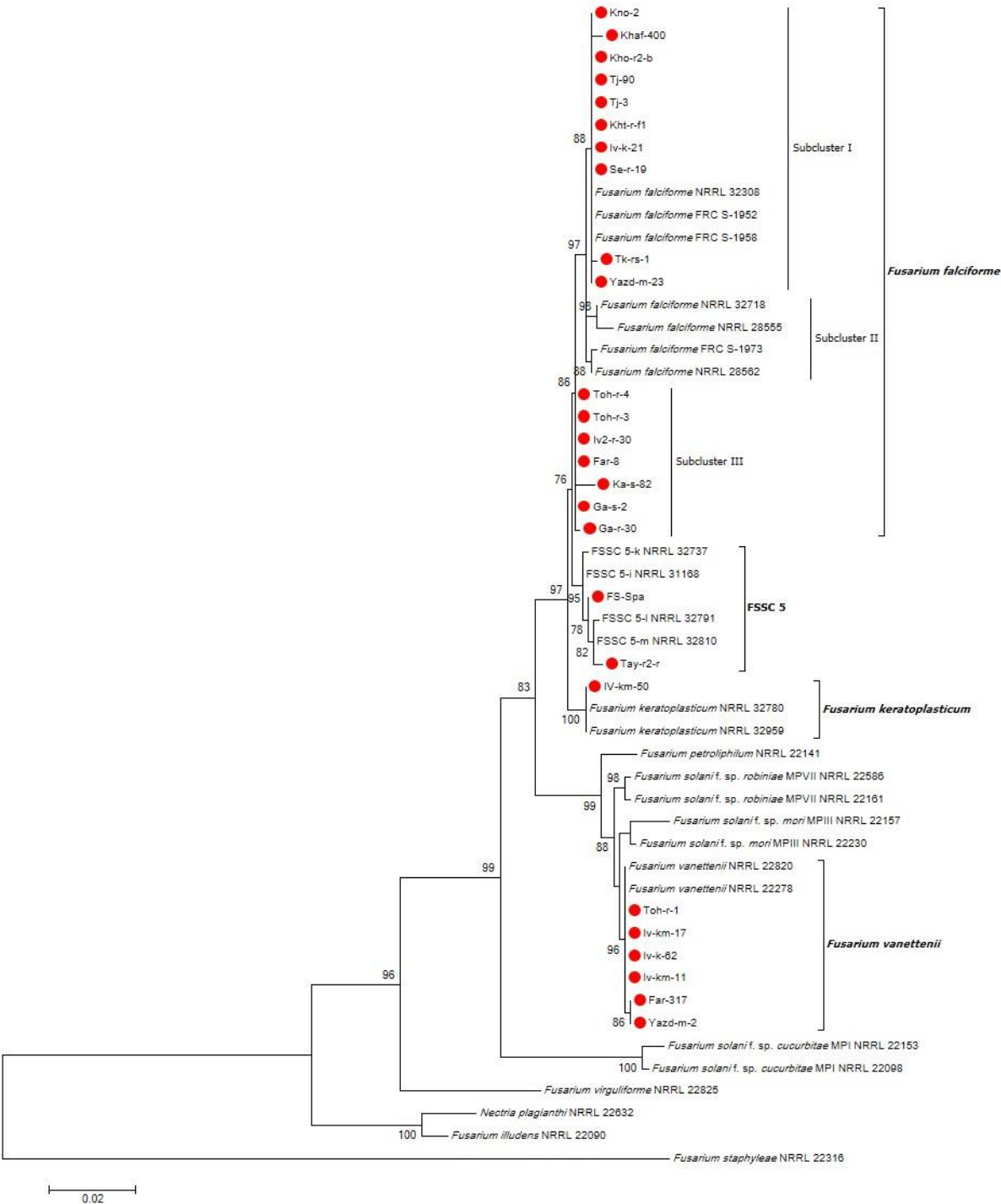

Supplement: Supplementary file 1 [file jof-09-00486-s001.zip › Figure S3.pdf]

Figure S5

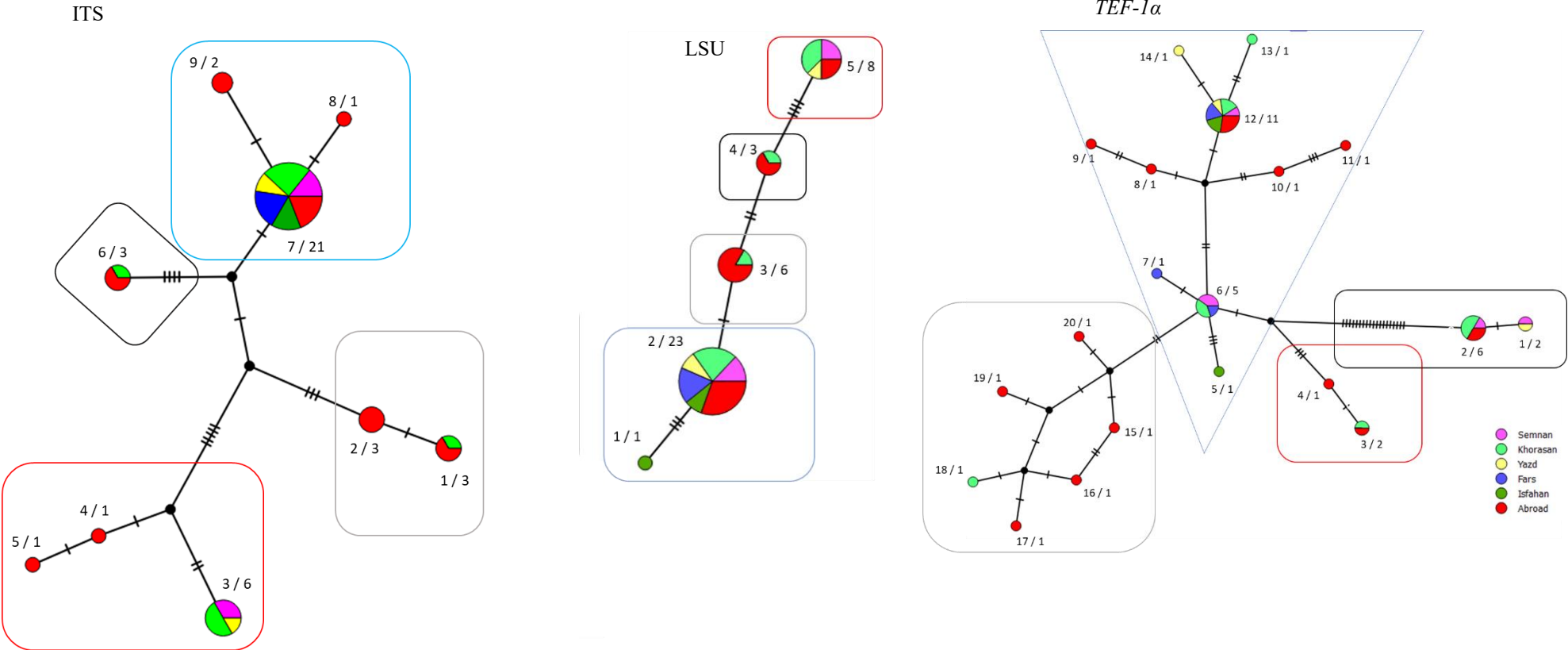

Supplement: Supplementary file 1 [file jof-09-00486-s001.zip › Figure S5.pdf]
